# How equity in cancer services has been defined and measured, and why it matters: a scoping review of Universal Health Coverage systems

**DOI:** 10.1080/16549716.2026.2733814

**Published:** 2026-09-16

**Authors:** Brighid Scanlon, Natasha Roberts, David Wyld, Jo Durham, Suping Ling, Bernard Rachet

**Affiliations:** a Cancer Care Services, Royal Brisbane and Women’s Hospital, Queensland, Australia; b Faculty of Health, Queensland University of Technology, Queensland, Australia; c Faculty of Medicine, University of Queensland, Queensland, Australia; d Surgical Treatment and Rehabilitation Services, STARS Education and Research Alliance, Herston, Queensland, Australia; e Inequalities in Cancer Outcomes Network, London School of Hygiene and Tropical Medicine, London, UK

**Keywords:** Cancer, health equity, epidemiology, social determinants, Universal Health Coverage

## Abstract

Cancer equity is a global priority, particularly within Universal Health Coverage systems that are committed to reducing disparities in access and outcomes. However, practical barriers to addressing inequities persist, such as varying definitions and clinical measurements. This inconsistency leads to a reliance on data that does not accurately reflect existing inequities or progress towards equitable care. Commonly reported measures are often aggregate, population-level indicators, such as cancer incidence and mortality, which fall outside the scope of health services and clinicians to influence. A detailed protocol and search strategy were developed a priori, following the Joanna Briggs Institute Reviewer’s Manual. Systematic searching was conducted via PudMed Central, CINAHL EBSCO and Cochrane, resulting in 53 studies meeting the selection criteria for analysis. Only eight (*n* = 8) studies offered definitions of equity, commonly emphasising accessible health services that support optimal health outcomes and the elimination of financial barriers. Seven (*n* = 7) main equity-related exposures or populations were associated with experiencing cancer inequities: ethnic minority or migrant populations, low socio-economic status, geographic location or remoteness, experiencing severe mental illness, low health literacy, high comorbidities, and those with a disability. A total of fifty-nine (*n* = 59) individual measures were utilised to demonstrate inequities. The absence of standardised, reproducible data undermines cancer equity efforts, as ad hoc reporting can misrepresent disparities or perpetuate the statistical invisibility of disadvantaged populations. This review aimed to summarise how equity has been defined and measured within UHC contexts, to inform the development of intentional and validated indicators for measuring and addressing cancer inequities.

## Paper Context

- **Main findings:** There is substantial variation in how equity in cancer is defined and measured within UHC contexts.
- **Added knowledge:** This review provides a comprehensive synthesis of cancer equity definitions, key populations experiencing inequities and measurement approaches used within UHC contexts.
- **Global health impact for policy and action:** The development of a standardised definition for cancer equity and a core set of indicators will support global action on cancer inequities by enabling health services, researchers, and policymakers to consistently identify, measure, and address inequities.

## Background

Growing global concern about health inequities has prompted many countries to prioritise reducing disparities as part of efforts to achieve Universal Health Coverage (UHC) [1,2]. The World Health Organization (WHO) defines UHC as ‘*all people have access to the full range of quality health services they need, when and where they need them, without financial hardship’* [3]. Achieving equitable health outcomes is increasingly recognised as both a moral imperative and a strategic necessity for sustainable development and global health security [4]. Health equity has been defined as the *‘principle underlying a commitment to reduce and ultimately, eliminate disparities in health and in its determinants, including social determinants’* [5]. Central to this definition is the recognition that health inequities are avoidable, unfair, socially produced, and reflective of one’s social positioning [5–7]. As such, achieving health equity is predicated on the ability to accurately measure, monitor, and act on inequities, and their determinants [8,9]. In contrast, health inequalities refer to measurable differences in health between population groups, without necessarily implying that these differences are avoidable or unfair [10,11]. Health disparities describe differences in health that are closely associated with social, economic or environmental disadvantage and can be used to quantify the magnitude of inequities between populations [10]. Despite commitments, health inequities persist throughout UHC contexts, which continue to report significant disparities in healthcare access, treatment outcomes and quality of care [12–16]. These inequities are exacerbated by resource limitations, governance issues and ongoing impacts from COVID-19 and international conflicts [17,18]. These factors have contributed to a stagnation of health outcomes and a decline in overall wellbeing and life expectancy, as demonstrated in the United Kingdom and Europe [19].

Research from Australia and England has demonstrated significant cancer disparities, particularly affecting those who are socio-economically disadvantaged [20–23]. Several measures have been utilised within the literature to identify and describe inequities within UHC contexts, such as lower participation in routine screening [15,24], suboptimal diagnostic pathways [14,25], and shorter survival [26,27]. Whilst useful, there is a current lack of standardised, evidence-based measures (indicators) that enable health services to identify and monitor inequities in cancer. Presently, most measures are reported by researchers and are not utilised by clinical health providers. Current measures are primarily determined by data availability, rather than reflecting the needs of patients, clinicians, or health systems [28]. Available measures are also largely generated *a posteriori*, reflecting late consequences of existing inequities. Importantly, many of the available measures are aggregate, population-level indicators, such as cancer incidence and mortality, which fall outside the scope of health services and clinicians to directly influence or change. These limitations may delay the realisation of health equity, as interventions derived from these measures may lack an evidence base and may misrepresent or omit disadvantaged populations within health data and interventions [29,30].

Within UHC contexts such as England, persistent socioeconomic inequalities in cancer survival have been consistently demonstrated [26,31,32]. Similarly, Canadian research has reported reduced screening behaviour, follow-up of abnormal findings, shorter survival, and lower quality care in ‘ethnic minority’ populations [24,33]. In Australia, previous research has identified several key cancer equity indicators. This included screening participation, time from diagnosis to commencement of treatment, clinical trials participation, and time from diagnosis to death [28,34]. Similarly, the English National Health Service (NHS) has performance indicators, such as cancer waiting time targets [35]. The NHS also has a set of general health equity indicators aimed at reducing preventable emergency department presentations arising from social inequality, such as general practitioner ratios [36]. While current measures provide valuable information, there is a critical need for standardised indicators which have been designed and evaluated as intentional measures of equity for clinical cancer services [8]. The current lack of reproducible, epidemiological data threatens health equity as it can misrepresent those experiencing health inequities, or perpetuate the statistical invisibility of disadvantaged populations [37]. Therefore, this review aims to summarise how equity has been defined and measured in cancer services in UHC contexts, to inform the development of a core set of clinical cancer equity indicators which enable measurement, monitoring, and action on inequities at the health service, or hospital level [8].

### Literature review process

A scoping review was identified as the most appropriate method, as it enabled a comprehensive review of the current literature. This allowed for the exploration of definitions and measures of (in)equity in cancer, including the identification of commonly reported measures for clinical outcomes, access, and treatment quality. A review protocol and search strategy were developed a priori in accordance with the Joanna Briggs Institute methodology for scoping reviews [38] but was not registered.

The primary review question was:

1. How has equity in cancer care been defined and measured in clinical health services research?

Secondary questions of interest were:

1. What inequities have been demonstrated?
2. What populations are experiencing these inequities?

This review follows the guidelines provided by the Joanna Briggs Institute (JBI) Reviewers Manual [39] and the PRISMA-ScR Guidelines (2020) [38].

To be included in this review papers were required to:

1. Demonstrate cancer inequities in diagnosis, outcomes, access, or treatment quality
2. Be positioned within secondary or tertiary-level health services
3. Include data from countries that have established or are progressing towards UHC, consistent with the WHO definition [3]
4. Be published between the years 2014–2025
5. Any language

Papers were excluded if they:

1. Include non-malignant diseases or conditions
2. Reported on Non-UHC contexts, such as Medicare, Medicaid, or TRICARE in the United States
3. Reported on those aged under 18 years
4. Report on cancer inequities in primary care services
5. Reported on inequities demonstrated via secondary sources
6. Were conference abstracts and case studies
7. Published prior to 2014

Demonstrated cancer inequities were defined as statistically significant differences between population groups in cancer diagnosis, outcomes, access, or quality of care. The review summarised how cancer equity has been conceptualised and measured in literature and therefore included papers reporting health differences, inequities, inequalities or disparities. Studies that did not demonstrate or measure cancer (in)equities were excluded. Countries were eligible if they had established or were progressing towards UHC, consistent with the WHO definition. UHC was assessed using the WHO UHC Service Coverage Index (Sustainable Development Goal 3.8.1) and consideration of progress towards financial protection (Sustainable Development Goal 3.8.2) [3]. Data from non-UHC contexts were excluded, such as Medicare, Medicaid or Tricare in the United States of America. Studies which exclusively examined subpopulations covered by Universal Health Coverage schemes, such as the United States Department of Veterans Affairs (VA), were included due to their alignment with the WHO UHC definition [3]. Primary qualitative, quantitative, and mixed-method studies were included for comprehensiveness. Secondary sources, such as systematic reviews and literature reviews, were not included. Grey literature, such as government reports and clinical guideline databases, were not included [40]. No studies were excluded due to quality, as per the purpose of scoping reviews.

A comprehensive search strategy was developed a priori and first implemented on 1 November 2024, with the search updated on 17 November 2025 to ensure currency. Searches were conducted in PubMed, CINAHL (EBSCO), and the Cochrane Library. The search strategy combined four key concepts: health equity, cancer/oncology, measurement or indicators, and clinical study designs, using controlled vocabulary (including MeSH) and free-text terms combined with Boolean operators (AND/OR). UHC was not included as a search concept but was applied as an eligibility criterion during screening, based on the WHO definition and assessment of countries’ progress towards UHC [3]. Full database-specific search strategies are provided in Supplementary File 1. The search produced 2948 results, which were transported into EndNote X9 to remove duplicates and then screened and analysed in Covidence [41]. Two independent reviewers screened titles, abstracts and full-text articles (BS and NR), with discrepancies resolved by discussion, or by a third independent reviewer (BR), if required. Studies published in any language were eligible for inclusion. Of the small number of non-English publications identified, all had an English-language version available through the journal, ranging from an English title and abstract to a full-text English version. These English-language versions were used for screening, and no non-English publication was unavailable for full-text screening. Citation and reference-list checking were not performed. A formal critical appraisal and risk of bias assessment were not conducted, as the purpose of this scoping review was to comprehensively map all available definitions and measurements of cancer equity utilised within the literature, rather than assess the methodological quality of individual studies.

### Results

**Figure 1.**
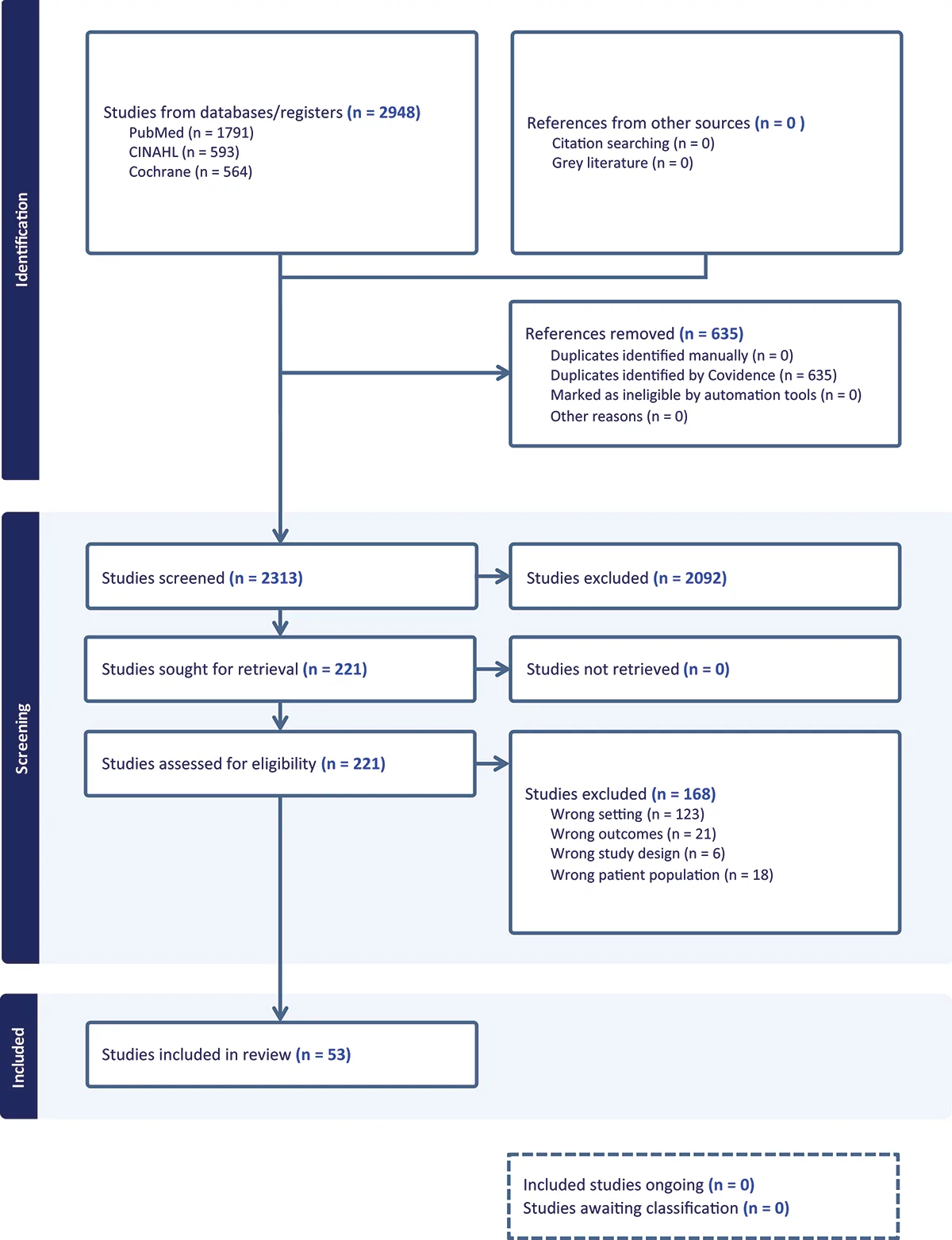
PRISMA flowchart.

The search strategy identified a total of 2948 records from PubMed (1791), CINAHL (593) and Cochrane (564) databases. Figure 1 displays the screening process. Duplicates were removed using Covidence, leaving 2313 initial studies. Title and abstract screening excluded 2092 studies and a further 168 were excluded during full-text screening. A total of 53 studies met the inclusion criteria and were included in the final analysis. The 53 included studies were conducted in Canada (*n* = 8), France (*n* = 7), England (*n* = 7), Australia (*n* = 6), the United States Veterans Affairs (*n* = 6), South Korea (*n* = 3), Brazil (*n* = 2), China (*n* = 2), Israel (*n* = 2), New Zealand (*n* = 2) and one study from Iran, Belgium, Argentina, Japan, Singapore, Sweden, Italy and South Africa. The majority (92%) were cohort studies. Most studies (73%) reported on data from population-based cancer databases or registries. The most reported cancer types were breast (*n* = 14), multiple cancer types (*n* = 10), lung (*n* = 7), colorectal (*n* = 7). Sample sizes ranged from 252–7,889,984.

### Definition of equity

Eight studies provided an explicit definition or conceptualisation of equity, presented in Table 1 [28,41–47]. Definitions varied substantially in their conceptual emphasis, with access the most frequently identified dimension, followed by health outcomes and affordability. Three studies conceptualised equity primarily in terms of access to timely and appropriate cancer care [42–45], while two incorporated financial or affordability considerations as a component of equitable access [44,45]. Other conceptualisations focused on the relationship between social and structural conditions and health outcomes, including the social gradient of health [43] and social determinants of health [48]. One study explicitly incorporated fairness and avoidability into its definition of health inequity [28], while another identified healthcare practitioner bias and structural barriers as contributors to inequitable access [47]. Concepts relating to need were less consistently incorporated, appearing explicitly in one definition [42].

**Table 1.** Definition and conceptualisations of equity.

Author	Definition/conceptualisation of equity	Equity concepts/dimensions addressed	Conceptual model/theoretical basis	Operational measure/s
Scanlon (2023)	Systematic, unfair and avoidable differences in opportunities to attain optimal health and health outcomes	Fairness; avoidability; access/opportunity; outcomes	Braveman conceptualisation of health equity/inequity; no formal equity measurement framework reported	Time from diagnosis to commencement of first treatment (months) Declining recommended chemotherapy (Y/N) Disease progression (Y/N) Number of unplanned admissions (*n*) Length of unplanned admissions (days) Failure to attend appointments (*n*)
Saito et al (2021)	Optimal healthcare access: (i.e. timely and appropriate cancer diagnosis and treatment, as well as the assessment of patient comorbidities)	Access; need	No formal equity framework reported	Emergency presentation (Y/N) Mode of surgery (Elective/Urgent)
Rylands (2016)	Social gradient in head and neck cancer: correlation between higher social deprivation and worse outcomes in oral cancer. Patients living in areas of higher deprivation have higher incidence of oral cancer and worse survival as well as a worse quality of life.	Outcomes	Social gradient of health; social determinants of health	Quality of life (UW-QOL social-emotional function subscale)
Rasmussen et al (2023)	Equal-access health care systems: strive to address financial barriers to health care and are generally thought to mitigate, although not eliminate, health disparities	Access; outcomes; affordability	Equal-access healthcare conceptualisation; no formal equity framework reported	Time to metastasis from progression to non-metastatic castration-resistant prostate cancer (years) Time to death from progression to non-metastatic castration-resistant prostate cancer (years) Comorbidity index (Mild, Moderate, Severe) PSA Doubling Time (>10 months/<10 months)
Recondo et al (2019)	Universal coverage: including access to public hospitals and primary care facilities to all individuals free of charge	Access; affordability	Universal coverage conceptualisation; no formal equity framework reported	Stage at diagnosis (Stage 1–4) Performance status (ECOG) Time from diagnosis to treatment commencement (days)
Malhotra et al (2020)	Universal Health Coverage: access to affordable palliative care to reduce inequities in “total pain” and suffering	Access; affordability; outcomes	Universal Health Coverage conceptualisation; no formal equity framework reported	Physical and functional wellbeing (FACT-G V4) Pain severity and interference (Brief Pain Inventory) Severity of other symptoms (FACIT-Pal) Psychological outcomes (Hospital Anxiety and Depression scale) Social wellbeing (FACT-G) Spiritual outcomes (FACIT-Spiritual) Perceived health care quality (Consumer Assessment of Healthcare Providers and Systems (adapted)
Choi et al (2025)	Barriers to timely and appropriate cancer diagnosis and care can result in poorer prognosis and higher mortality. Barriers include physical accessibility, healthcare practitioner bias and socioeconomic disadvantage, which hinder equitable access to timely screening, diagnosis and treatment.	Access; fairness; outcomes	Disability-related barriers conceptualisation; no formal equity framework reported	Stage at diagnosis (localised, locoregional, distant, unknown) Receipt of standard treatments: (surgery Y/N, chemotherapy Y/N, Radiotherapy Y/N) All-cause mortality (%) 5-year survival
Bondzi-Simpson et al (2025)	Social determinants of health: social structures and economic systems can result in unequal access to power and resources that influence health and health outcomes.	Access; outcomes	Social determinants of health conceptualisation; no formal equity framework reported	Receipt of chemotherapy (within 3 months of surgery)

The conceptual underpinnings of the definitions also varied. These included Braveman’s conceptualisation of health equity/inequity [28], the social gradient of health [43], universal health coverage [44,45], equal-access healthcare [46], and social determinants of health [48]. None of the eight studies reported applying a formal, established framework specifically designed to conceptualise and measure equity in cancer services. Rather, equity was operationalised through diverse measures of healthcare access, treatment utilisation, timeliness, affordability and health outcomes across different population groups.

### Populations associated with inequities

This review identified seven (*n* = 7) main equity-related exposures or populations associated with experiencing cancer inequities, presented in Table 2. These included being part of an ethnic minority or migrant population (19%) [28,44,48–55], being of low socio-economic status (47%) [42,43,46,48,52,53,56–72], geographic location or remoteness (30%) [51,53,60,65,73–83], experiencing mental illness (9%) [84–88], having low health literacy (2%) [89], high comorbidities (4%) [44,70], or those with disabilities (2%) [47]. Each of these populations were associated with a range of clinical outcome measures. The measurement of each exposure or population differed across studies. Terminology varied considerably across studies, with some reporting ‘ethnicity’ [48–51,54], others ‘race’ [53], some using the terms interchangeably [44,56], and others reporting them collectively as ‘race/ethnicity’ [55]. These data were largely reported by categorising populations into multiple main ethnic groups [44,49–52,55]. However, some studies highlighted disparities by using a binary classification, such as ‘African American vs. White’ [49,54,56]. Similarly, the study assessing migrant status was reported as a binary ‘Australian-born vs. CALD migrant’, defined by place of birth and first language spoken [28]. Where reported, race and ethnicity data were predominantly derived from self-reported information recorded in electronic medical records, cancer registries, administrative databases, or single cancer centre records. Socio-economic status, also reported as ‘deprivation’ was measured using a variety of methods, including both ad hoc and formalised measures. Ad hoc measures included employment [57], supplementary insurance [57], house ownership [57], level of education [58,66,71], household composition [58], housing conditions [58], annual household income [63], individual-level income [66], self-reported financial difficulties [46,66] and annual per capita household expenditure [69]. Formalised SES measures included Reunion Deprivation Index [90], Social Deprivation Index [59], Index of Multiple Deprivation [43,52,64] and Ontario Marginalisation Index [61]. Geographic location was measured using remoteness scales, including Metropolitan, Inner Regional, Rural [65,74,80], Intermediate Regions of Urban Articulation [75], Urban vs Rural [78,82] and Capital city vs Non-Capital city [76]. Additionally, travel time (in minutes) [79,81], distance from the treating hospital (in kilometers) [51,53,83], and specialist service density (expressed as a ratio) [75] were also utilised. Mental illness exposure was measured as having a diagnosis or history of severe psychiatric illness [84–88]. Health literacy was measured using the Single Item Literacy Screener [89] and the Test of Functional Health Literacy [89] in Adults. Comorbidities were measured using the Charlson Comorbidity Index [44,70].

**Table 2.** Taxonomy of measurement for cancer equity-related exposures/stratifiers.

Equity-related Exposure/ Stratifier*	Conceptual Domains**	Measurement Approaches
*Ethnic minority/migrant status*	Sociocultural identity Country of birth/Migration history	Individual self-reported: Self-identified race/ethnicity; ethnic group classification; country of birth; migration history Administrative/medical record/registry database: Race/ethnicity recorded in electronic medical records or cancer registries Categorical approaches: Multi-category ethnic classifications; binary ethnic comparisons; binary migrant status
*Socio-economic status (SES)/ Deprivation*	Formal deprivation indices (area-level) Individual-level SES indicators Mixed/ad hoc SES constructs	Area-level (Formal Indices): Index of Multiple Deprivation; Social Deprivation Index; Reunion Deprivation Index; Ontario Marginalisation Index, typically categorised into quintiles/quartiles Individual components (Self-reported): Employment; education; household income; individual income; household composition; housing conditions; home ownership; financial difficulties; expenditure Administratively reported data: Supplementary/private health insurance; employment Composite: Study-specific SES scores (individually defined)
*Geographic Location/ Remoteness*	Remoteness classification Geographic accessibility Healthcare service availability	Area-level: Metropolitan/urban, regional and rural classifications; capital city/non-capital city; Intermediate Regions of Urban Articulation (derived from postcode or residential location recorded in medical/administrative records) Geographic measures: Travel time to treatment (minutes); distance to hospital (km) Health-system: Specialist density ratio (per population)
*Mental Illness*	● Clinical psychiatric diagnosis ● Psychiatric history	Administrative/medical record: Clinical psychiatric diagnosis; history of severe psychiatric illness; psychiatric episodes; psychiatric hospitalisation Categorical approaches: Diagnosis/history of schizophrenia, bipolar disorder, major psychotic disorders and other severe psychiatric conditions
*Health Literacy*	● Functional health literacy ● Proxy literacy indicators	Individual self-reported/assessed: Single Item Literacy Screener (SILS); Test of Functional Health Literacy in Adults (TOFHLA)
*Comorbidities*	● Clinical multimorbidity burden	Administrative/medical record: Charlson Comorbidity Index (total score or grouped (0/1/≥2)
*Disability*	● Functional limitation ● Self-reported disability status	Individual self-reported: Self-reported disability status; functional limitations Administrative/medical record: Disability status where recorded

* Equity-related exposures/stratifiers refer to demographic, socioeconomic, geographic, clinical or health-system characteristics examined as exposures or used to stratify populations when assessing differences in cancer care, access or outcomes. ** Conceptual domains refer to the specific constructs or characteristics within each equity-related exposure/stratifier that were assessed across the included studies.

### Clinical outcomes associated with inequity

Clinical outcome measures associated with inequities were reported along the cancer continuum, including diagnosis, treatment, quality of care, service utilisation and survival measures. There was some variation in how these outcomes were measured, with all clinical outcome measures presented in Table 3. Diagnostic measures included patient age [49,50,56,91], stage [45,47,54,58,62,75,78,83,87,90,91], and tumour size at diagnosis [49], delays to diagnosis and referrals [71,88], and higher emergency diagnoses (receiving diagnosis during or after emergency presentation) [88]. Treatment measures included delays to treatment commencement (time from diagnosis to treatment commencement) [28,45,51,53,56,73,74,84], less treatment uptake (receipt of treatment) [47,48,50,61,62,73,74,87,92], lower access to novel therapies [64] and lower participation in clinical trials [76,79,89]. Care quality was reported as lower quality of life [43,66] and self-reported health [57,58], less multidisciplinary team reviews [74], lower symptom assessment [85] and higher symptom burden [46,85,88]. Service utilisation measures included higher unplanned admissions [28], longer length of admissions [28], higher emergency department presentations [28,42,70,71,81,86] and higher admissions to the intensive care unit [86]. A range of survival measures were used, including higher disease progression [28,44] and recurrence [50,86,91], lower disease-free survival [63,82], shorter time from diagnosis to death [44], higher 30-day mortality [65], higher in-hospital deaths [70] and higher all-cause [47,87] and cancer-specific mortality [87,92].

**Table 3.** Taxonomy of measurement for cancer equity outcomes.

Outcome categories	Conceptual Domains	Measurement Approaches
*Diagnosis*	*Clinical tumour characteristics* *Cancer staging* *Diagnostic pathway* *Disease burden at diagnosis*	1. **Demographics** Age at diagnosis (years) Diagnosis within an Emergency Department (Y/N) 2. **Tumour morphology/pathology** Tumour size (cm) Tumour grade (Grade 1–3) 3. **TNM cancer staging** Tumour stage (T1–4) Nodal stage (0–3) Stage at presentation (T1–4) Stage at dx (Stage T1–4) Late stage at diagnosis (Proportion of T3/T4) 4. **Other cancer staging** Stage at dx (localised, locoregional, distant, unknown) Stage at dx (early/advanced/unknown) Stage at dx (localised, regional, distant) 5. **Disease burden/biomarkers** PSA levels Residual metastatic burden (RBC 0–3) Proportion of late stage at diagnosis per IRUA Metastasis at dx (Y/N) 6. **Diagnostic timelines** Fast-track specialist referral (2-week wait) Time from colonoscopy to diagnosis Delayed diagnosis (first hospitalisation preceded by ED visit) Secondary care diagnostic interval (days) Whole diagnostic interval (days) Access to advanced drug therapies for lung cancer within 2 years post dx
*Treatment*	*Treatment receipt/uptake* *Clinical trial and advanced therapy participation* *Stage-appropriate/guideline-based care* *Treatment timing/intervals* *Medication adherence and persistence*	1. **Treatment type (Binary measures)** Receipt of surgery (Y/N) Receipt of chemotherapy (Y/N) Receipt of radiotherapy (Y/N) Receipt of treatment (surgery, CTX, XRT; Y/N) Receipt of imaging (Y/N) Receipt of EMR treatment (Y/N) Receipt of standard treatments (surgery, CTX, XRT; Y/N) Receipt of adjuvant therapies Non-receipt of surgical resection Non-receipt of adjuvant chemotherapy Non-receipt of adjuvant radiation Receipt of PET imaging (<6 months pre-surgery) Receipt of PFTs (<6 months pre-surgery) Utilisation of novel therapies (Y/N) Utilisation of novel anti-cancer therapies (molecular targeted therapy, biologics, immunotherapy) Declining recommended chemotherapy (Y/N) Prescription frequency of anticancer drugs (FICHCOMP) 2. **Guideline-Concordance Metrics** Receipt of stage-appropriate treatment (guideline-based) Stage-appropriate treatment (per guidelines) Mode of surgery (Elective/Urgent) Access to MIS vs open hysterectomy 3. **Treatment timelines** Initiated treatment within 60 days of diagnosis Time to treatment commencement (<30 days vs > 30 days) Time from diagnosis to first treatment (days/months) Time from diagnosis to commencement of first treatment (months) Time to surgery (difference between diagnosis and first surgery) Time from dx to surgery (days) Timely surgery (definitive surgery within 12 weeks of radiographic diagnosis) Whole interval (days) Time to distant metastasis following prostate cancer dx Time from progression to non-metastatic CRPC to metastasis Time from progression to non-metastatic CRPC to death Receipt of CTX within 3 months of surgery Delay in adjuvant treatment (>90 days radiotherapy, >60 days chemotherapy) Treatment interval (days) 4. **Medication** Persistence (medication taken for intended timeframe) Adherence (MPR <80%) 5. **Clinical trial/s** Clinical trial invitations (self-reported) Clinical trial participation Trial geographical equity index (TGEI) Ratio of clinical trials open to non-capital vs capital cities (Trial Geographical Equity Index, TGEI)
*Quality of care*	*Patient-reported outcomes Symptom burden* *Functional status* *Clinical process measures*	1. **Patient reported measures** Self-reported health questionnaire Social-emotional (UW-QOL social-emotional subscale) Quality of life (QLQ-C30) score at diagnosis and 2-year follow up Physical and functional wellbeing (FACT-G V4) Psychological outcomes: Hospital Anxiety and Depression Scale Symptom burden: (ESAS-r) Pain severity: Brief Pain Inventory (BPI) Severity of other symptoms (FACIT-Pal) Health utility (Health Utilities Index Mark 3) Social wellbeing (FACT-G) Spiritual outcome (FACIT-Spiritual) Financial toxicity (FIT Tool) Consumer Assessment of Healthcare Providers and Systems (CAHPS *adapted)* Clinical assessments Performance status: ECOG Stage-appropriate treatment adherence (per guidelines) Appropriate smoking management (smokers receiving cessation therapy within 12 months pre-surgery) 2. **Process Quality Metrics** Immediate reconstruction rate (%) Postoperative complications (Y/N) Inpatient complications (not further defined) Had MDT review (Y/N) Receipt of PET imaging (<6 months pre-surgery) Receipt of PFTs (<6 months pre-surgery)
*Service utilisation*	*Unplanned, urgent, or emergency use of hospital services* *Outpatient and inpatient service use* *Palliative care access* *Specialist services and advanced therapies*	1. **Acute Care Utilisation (Binary/Discrete measures)** Number of unplanned admissions (n) Length of unplanned admissions (days) ED visits (n) ED visits in last year of life (n) ICU admissions in last year of life (n) High-intensity palliative care indicators (intra-hospital chemo last 14 days, mechanical ventilation, transfusions, surgery, imaging/endoscopy, ≥1 ED/ICU/acute care admission in last 31 days) Hospital as place of death (n) Failure to attend appointments (n) Receipt of specialist palliative care (Y/N) Access to palliative care in past 31 days (Y/N) Outpatient chemotherapy in last year of life (n) Outpatient radiotherapy in last year of life (n) 2. **Acute Care Utilisation (Interval measures)** Inpatient service use (%) Outpatient service use (%) Neighbourhood rates of laparoscopy Duration from first palliative care to death (days)
*Survival measures*	*Cancer recurrence and progression* *Disease-free survival* *Metastasis-free survival* *Overall survival and mortality* *Postoperative and short-term mortality*	1. **Recurrence/Progression Measures (Binary)** Cancer recurrence (Y/N) Disease progression (Y/N) 2. **Overall Recurrence (Population-level)** Overall recurrence (3-year & 5-year) 3. **Formal Recurrence/Progression-Free Survival Endpoints** Relapse-free survival Disease-free survival (DFS) Distant metastasis-free survival 4. **Time-to-event measures** Time to metastasis from progression to non-metastatic CRPC (years) Time to distant metastasis following prostate cancer diagnosis (days) 5. **Population-level Survival Measures** Overall survival (%) 5-year survival Cancer-specific survival All-cause mortality (%) Postoperative mortality (during intervention or within 90 days) 30-day mortality Mortality within 2 years Cancer-specific survival

### Function and impact of reported equity measures

Most (92%) included studies were observational cohort designs, primarily focused on investigating and quantifying previously unidentified health inequities. These studies commonly compared epidemiological, clinical, pathological, and health service use characteristics across various population groups. In some cases, research was conducted in response to existing disparities, aiming to explore a range of sociodemographic variables to further understand factors contributing to these inequities. Most cohort studies were conducted retrospectively and relied on routinely collected data from population-based cancer registries and administrative health databases. Consequently, the measures used to identify and describe inequities were largely determined by data availability. Most measures reflected downstream clinical outcomes, such as stage at diagnosis [45,47,54,58,62,75,78,83,87,90,91] or disease-free survival [63,82], or patterns of health service utilisation, such as treatment uptake [47,48,50,61,62,73,74,87,92] or unplanned hospital admissions [28]. Relatively few studies established a functional link between these outcomes and upstream social or structural determinants of health. The clinical and service-based measures largely served a descriptive purpose, by highlighting existing disparities, but few critically evaluated their utility as tools for monitoring and acting on health inequities. Many studies underscored the significance of their findings in promoting equitable care and informing policy development, only twelve (23%) provided actionable recommendations at the health system or hospital level to address the observed inequities [28,42,46,48,52,61,68,71–73,85,89].

## Discussion

This review provides a comprehensive synthesis of how equity has been defined and measured within clinical cancer services in the context of UHC. A range of measures were identified across the cancer continuum, including the dimensions of diagnosis, treatment, care quality, service utilisation and survival. Key findings include the lack of consistent or clearly articulated definitions of equity across literature, substantial variation in the exposure and outcome measures used to assess equity, and limited critical evaluation of the effectiveness of the measures employed, including a lack of actionable recommendations for health services. Importantly, this review demonstrates that cancer inequities persist globally, even in the context of UHC, highlighting the urgent need for the development and evaluation of equity indicators that enable actionable solutions for health services.

### Lack of conceptual framework

Only eight of the included studies provided a definition or conceptual framework associated with health equity [28,41–47], with most highlighting optimal health outcomes and a lack of financial barriers to care. Despite the increasing emphasis on equity in health policy and service delivery, this review reveals persistent conceptual ambiguity within the published literature [9,93,94]. This raises a fundamental concern regarding the conceptual basis for what is measured and subsequently characterised as cancer equity. A range of terms, such as inequity, inequality or disparity, were used to describe differences in treatment, healthcare utilisation or health outcomes between population groups, without establishing whether these differences were unfair, avoidable or socially produced. As Braveman, a leading health equity scholar, argues, *‘health disparity’* was not intended to encompass *‘all possible health differences among all possible groups of people’* [5]. As such, not all observed differences constitute inequities, as some may be unrelated to unfair or avoidable disadvantage. Consequently, observed differences between population groups require further examination and explicit terminology to determine whether they represent cancer inequities.

International literature reports a clear need for explicit definitions and robust measures in health equity research, moving beyond reliance on softened terminology that obscures structural disadvantage, and proxy measures that interpret differences between population groups as inequities without establishing whether they are unfair, avoidable, or socially produced [5,95]. For example, using phrases such as ‘differences in treatment receipt’ rather than ‘inequitable access to treatment’ avoids explicitly identifying differences as unfair, avoidable or socially produced, and therefore whether they represent inequities or inequalities. The use of this implicit or vague terminology obscures the nature and extent of inequities and risks underrepresenting populations experiencing structural disadvantage [29,30]. Moreover, this ambiguity undermines the interpretability and comparability of research findings across different contexts, thereby weakening the evidence base that informs policy and health system reform [96,97]. In order to operationalise health equity, it is essential that measurement approaches are underpinned by explicit definitions and conceptual frameworks and reflect the social and structural determinants that produce and reinforce inequities within health systems [98]. For clinical health services, this requires the development of robust measures and analytic approaches that can distinguish inequities from other forms of population difference, and are meaningful, actionable and capable of transparently monitoring health outcomes over time [99].

### High variation in outcome measures

An important finding of this review is the considerable variation in outcome measures used to examine cancer inequities. Most studies used established clinical indicators, including stage at diagnosis, disease-free survival, overall survival, treatment uptake and unplanned hospital admissions. These measures capture important aspects of clinical care and healthcare delivery and can identify differences in outcomes between population groups. However, the observation of a disparity does not, in itself, establish the presence of inequity. Determining whether observed differences are inequitable requires consideration of whether they are unfair, avoidable and socially produced, including the contribution of underlying social, structural and health-system factors [8,100]. Thus, while these clinical measures remain valuable for identifying populations experiencing poorer outcomes and informing intervention development, interpreting these differences as inequities requires an explicit equity lens and consideration of the social, structural and health-system factors that may produce and reinforce them [101,102].

### The need for functional equity metrics

Few studies assessed whether these clinical outcomes functioned as effective tools for monitoring progress toward equity goals. Only 12 (23%) provided any actionable recommendations at the clinical service level. Actionable recommendations identified across studies focused on embedding equity within routine hospital processes, including improved care coordination [74] and navigation [85], screening for social and financial needs [48,61,72], culturally responsive communication [28,48], health literacy support [89], and access to allied health, financial [46] and transportation assistance [73]. Other recommendations included strengthening equity-focused quality indicators [42,52], referral pathways [61], workforce capability [74] and digital models of care [68,85], highlighting opportunities for health services to translate equity measures into targeted, system-level action.

While many measures presented could detect statistically significant differences between populations, their practical utility in driving health service reform remains largely unexplored. Without rigorous evaluation, these metrics risk serving solely as descriptive indicators rather than drivers of structural change [103]. Measures that better address underlying social and structural determinants should capture the factors through which disadvantage influences cancer care, rather than simply stratifying clinical outcomes by population characteristics [96]. Systematically mapping available data and measures against policy frameworks can help identify gaps and support the development of actionable approaches for health services [104]. This current gap in equity measurement limits the capacity of health services to translate data on disparities into actionable strategies that address the underlying social and structural determinants of cancer inequities.

Health inequities represent more than mere differences in health outcomes between populations; they are avoidable, unfair, and unjust disparities shaped by social and structural determinants [5,6]. Importantly, this means that health inequities are not inevitable and that they can be influenced by structural change [7]. For clinical health services to meaningfully respond to cancer inequities, it is crucial that they move beyond the repurposing of traditional descriptive, clinical metrics. This requires explicit definitions, consistent terminology, reproducible measures and clear objectives that produce actionable recommendations for health services.

### Limitations

This review has several limitations. As a scoping review, the aim was to map the breadth of evidence on cancer equity definitions and measures rather than assess study quality; therefore, no critical appraisal was undertaken. The identified measures were drawn from studies with diverse definitions, populations, and healthcare contexts, which may have influenced how cancer inequities were conceptualised and reported. Additionally, only studies from Universal Health Coverage contexts were included, limiting the generalisability of findings to other healthcare settings. Consequently, the findings should be interpreted as a summary of existing definitions and measures, rather than as an assessment of their validity as indicators of cancer equity.

## Conclusions

In conclusion, this review highlights the high variation in the definitions and instruments used to demonstrate inequities in cancer. Greater clarity is needed to distinguish measures of cancer inequity, which reflect unfair, avoidable and socially produced differences, from measures that simply describe inequalities between population groups. To meaningfully address cancer inequities, there is a critical need to develop a core set of standardised indicators that are intentionally designed, tested and evaluated as measures of equity within clinical cancer services.

## Supplementary Material

PRISMA_SCR_Checklist.pdf

Sup_File_1_Database_Searches.docx

Supplementary_File_2_Data_Extraction_Table.docx

## Data Availability

All articles included in this review are listed in Supplementary File 2. The search strategy is listed in Supplementary File 1. Any additional information generated during this study is available from the corresponding author upon reasonable request.
