# Supplementary material for: How equity in cancer services has been defined and measured, and why it matters: a scoping review of Universal Health Coverage systems
Source: Glob Health Action. 2026 Sep 16;19(1):2733814. doi: 10.1080/16549716.2026.2733814 (PMC13587566; doi:10.1080/16549716.2026.2733814)
Supplement: Supplementary file 2 [file ZGHA_A_2733814_SM3647.docx]

Supplementary File 2 (Data Extraction Table)

| **Author** | **Title** | **Year** | **Location** | **Setting** | **Study design** | **Sample size** | **Cancer Type** | **Definition of equity** | **Population experiencing inequity** | **Inequities demonstrated** | **Exposure** (Population and measurement) | **Outcome** (Clinical measurement) | **Actionable** recommendations for health services? |
| --- | --- | --- | --- | --- | --- | --- | --- | --- | --- | --- | --- | --- | --- |
| Shitrit et al | Epidemiological, clinical, and pathological characteristics of invasive breast cancer in Bedouin and Jewish women in southern Israel: a retrospective comparative study | 2023 | Israel | Teaching hospital | Cohort | 1705 | Breast | Not provided | Bedouin women | - Younger age at dx - Larger tumour size - Higher metastasis rate | Ethnicity: Bedouin women (vs Jewish women) | - Age at dx (years) - Tumour size (cm) - Tumour stage (T1-4) - Nodal Stage (0-3) - Metastasis (Y/N) | No |
| Taheri et al | Socioeconomic Inequalities in Metastasis, Recurrence, Stage, Grade, and Self-Rated Health of Gastrointestinal Cancer Patients | 2019 | Iran | Teaching hospital | Cohort | 409 | Gastrointestinal | Not provided | Low SES | - Higher grade at dx - Lower self-reported health | SES Level: (Employment, Supplementary insurance, house ownership) | - Tumour grade (1-3) - Self-reported Health Questionnaire | No |
| Hwang et al | Variations in breast cancer histology and treatment patterns between the major ethnic groups of South West Sydney | 2017 | Australia | Local Health District | Cohort | 1215 | Breast | Not provided | - Asian Pacific Islander (API) - White European | - Younger age at dx (API) - More likely to experience recurrence (White European) | Ethnicity:  (White Anglo-Saxon, White European, Asian/Pacific Islander, Middle Eastern, Other) | - Age at dx (years) - Receipt of radiation (Y/N) - Receipt of chemotherapy (Y/N) - Cancer recurrence (Y/N) | No |
| Saito et al | Exploring socioeconomic differences in surgery and in time to elective surgery for colon cancer in England: Population-based study | 2021 | England | Population-based electronic health records database | Cohort | 68,169 | Colon | Optimal healthcare access: (i.e. timely and appropriate cancer diagnosis and treatment, as well as the assessment of patient comorbidities) | SES (Most deprived) | - Higher proportion had emergency presentation - Higher proportion receive urgent surgery | SES/Deprivation: (Index of Multiple Deprivation Quintile: 1-5) | - Emergency presentation (Y/N) - Mode of surgery (Elective/Urgent) | - Emergency presentation and urgent surgery may be a more meaningful measure than current wait time targets |
| Rosskamp et al | Socio-Economic Position, Cancer Incidence and Stage at Diagnosis: A Nationwide Cohort Study in Belgium | 2021 | Belgium | Population-based registry | Cohort | 6,960,371 | Lung, Colon, Rectum, Head & Neck, Breast, Melanoma | Not provided | - Lower education - Living alone | - More likely to be diagnosed with unknown stage (lower education) - Higher unknown stage at dx (Living alone) - More advanced stage at diagnosis (Lower Education) | Socioeconomic position (Education, Household composition, Housing conditions, Self-reported health, region of residence) | - Stage at dx (Early/Advanced/Unknown) | No |
| Foley et al | Exploring the impact of remoteness on people with head and neck cancer: Utilisation of a state-wide dataset | 2023 | Australia | Population-based registry | Cohort | 1991 | Head & Neck | Not provided | Rural patients | - Less likely to have MDT review - Less likely to have treatment - More likely >30 days to treatment commencement | Remoteness (Metropolitan, Inner Regional, Rural) | - Had MDT review (Y/N) - Receipt of treatment (Had treatment/No treatment) - Time to treatment commencement (<30 days vs >30 days) | - Need to link Metropolitan treating team with care closer to home - Need for greater allied health support in the post-acute recovery phase |
| De Oliveira et al | Spatial distribution of advanced stage diagnosis and mortality of breast cancer: Socioeconomic and health service offer inequalities in Brazil | 2021 | Brazil | Population-based registry | Ecological | 195, 201 | Breast | Not provided | - Living in socioeconomically disadvantaged areas - Living in areas with density specialist services | - Higher proportions of late-stage dx | Geographical location (Intermediate Regions of Urban Articulation)  Density of specialist services (Ratio) | - Proportion of late stage at diagnosis, per IRUA (%) | No |
| Deneche et al | Socioeconomic inequalities in breast cancer survival in Reunion Island: The contribution of stage at diagnosis as a mediator | 2022 | Reunion Island (France) | Population-based registry | Cohort | 2892 | Breast | Not provided | Most deprived | - Higher advanced stage at dx | Deprivation (Reunion Deprivation Index) | - Stage at dx (localised, regional, distant) | No |
| Seneviratne et al | Ethnic differences in timely adjuvant chemotherapy and radiation therapy for breast cancer in New Zealand: a cohort study | 2014 | Waikato and Midlands (New Zealand) | Population-based registry | Cohort | 2848 | Breast | Not provided | - Indigenous Māori women - Rural women - Women treated in public hospitals - Women requiring surgical re-excision | - Delays in adjuvant treatment (Māori women, rural women, women in public hospitals, women requiring surgical re-excision) | Ethnicity (Maori, New Zealand European, Pacific, Other)    Distance from hospital (<10 km, 10-50km, 50-100km, >100km)    Facility type (Public, Private) | Delay in adjuvant treatment (>90 days radiotherapy, >60 days chemotherapy) | No |
| Scherpereel et al | Access to innovative drugs for metastatic lung cancer treatment in a French nationwide cohort: the TERRITOIRE study | 2018 | France | Population-based hospital database | Cohort | 11,486 | Lung | Not provided | Most socially deprived | - Poorer access to innovative drugs | Social Deprivation Index quartile (Most deprived, deprived, privileged, Most privileged) | - Prescription of anticancer drugs (Frequency of FICHCOMP drugs used) | No |
| Davis et al | Access to symptom screening and severe symptom risk among cancer patients with major mental illness | 2023 | Canada | Population-based electronic health records database | Cohort | 389,870 | All | Not provided | Patients with severe psychiatric illness | - Lower incidence of symptom screening - Higher symptom scores across all symptoms | Severe psychiatric illness | Symptom burden (ESAS-r) | - Symptom reporting via an app or visit with their regular mental health care team - Non-pharmacological symptom strategies - Integrating of mental and physical care needs via nurse navigator |
| Rylands et al | Outcomes by area of residence deprivation in a cohort of oral cancer  patients: Survival, health-related quality of life, and place of death | 2016 | England | Teaching hospital | Cohort | 553 | Oral | Social gradient in head and neck cancer: correlation between higher social deprivation and worse out-  comes in oral cancer. Patients living in areas of higher deprivation  have higher incidence of oral cancer and worse survival as  well as a worse quality of life. | Most deprived | - Worse quality of life | IMD 2010 quartile of residential area (1 most deprived-4 least deprived) | Quality of life (UW-QOL social-emotional function subscale) | No |
| Rasmussen et al | Survival Outcomes by Race and Ethnicity in Veterans with Nonmetastatic  Castration-Resistant Prostate Cancer | 2023 | United States | Veterans Affairs Cancer Registry | Cohort | 12,992 | Prostate | Equal-access health care systems: strive to address  financial barriers to health care and are generally thought to mitigate, although not eliminate, health  disparities | - White patients - Those with higher comorbidities - Those with PSA doubling time <10 months | - Shorter time to metastasis (White patients) - Shorter time to death (White patients) - Higher risk of death (Higher comorbidities, PSA doubling time (PSADT) <10 months) | - Ethnicity (Hispanic, Non-Hispanic white, Non-Hispanic Black, Other) - Comorbidities (CCI) - PSA (PSADT) | - Time to metastasis from progression to non-metastatic castration-resistant prostate cancer (years) - Time to death from progression to non-metastatic castration-resistant prostate cancer (years) - Comorbidity index (CCI) (Mild, Moderate, Severe) - PSA Doubling Time (PSADT) (> 10 months/<10 months) | No |
| Moss et al | Surgical trends, outcomes and  disparities in minimal invasive surgery  for patients with endometrial cancer in  England: a retrospective cohort study | 2020 | England | Population-based registry | Cohort | 35, 304 | Endometrium | Not provided | - Lowest socioeconomic group - Non-white ethnicity - Those living in regions with less MIS providers | - Lower MIS rates (Low SES, non-white, region with less MIS providers) | - Socioeconomic status (IMD) - Ethnicity (White, Asian, Black, Other) - Provider volume (High, Intermediate, Low, very low) | - Access to MIS vs open hysterectomy - Postoperative mortality (death during intervention or within 90 days) - Postoperative morbidity (gastrointestinal, wounds, infections, neurological, haemorrhage) during intervention admission and 90 days post | - Providing support and mentorship to low MIS providers - MIS rates for endometrial cancer is a proposed quality indicator |
| Recondo et al | Access of patients with breast and lung cancer  to chemotherapy treatment in public and  private hospitals in the city of Buenos Aires | 2019 | Argentina | Multiple teaching hospitals | Cohort | 281 | Breast, Lung | Universal coverage: including access to public hospi-  tals and primary care facilities to all individuals free of charge | Patients in public hospitals | - Higher stage at diagnosis - Lower performance status - Longer median time from diagnosis to treatment commencement (breast and lung) | - Public or private hospital | - Stage at diagnosis (1-4) - Performance status (ECOG) - Time from diagnosis to treatment commencement (days) | No |
| Jeevan | Reconstructive utilisation and outcomes following mastectomy surgery in women with breast cancer treated in England | 2020 | England | Population-based registry | Cohort | 55,297 | Breast | Not provided | - Socioeconomic deprivation - Regional variation (not further defined) | - More likely to have inpatient complications (Higher deprivation) - Regional variability in immediate breast reconstruction | - Socio-economic status - Geographical region | - Immediate reconstruction rate (%) - Inpatient complications (not further defined) | No |
| Jang et al | Regional disparities in the availability of cancer clinical trials in Korea | 2024 | South Korea | National database | Cohort | 9,424 | All | Not provided | Those in non-capital city regions | - Less access to clinical trials | - Region (capital city vs non-capital city) | - Ratio of clinical trials open to non-capital city relative to capital cities: Trial geographical equity index (TGEI) | No |
| Davis et al | Material Deprivation and Access to Cancer Care in a Universal Health Care System | 2020 | Canada | Population-based electronic health records database | Cohort | 39,319 | Gastrointestinal | Not provided | Those more materially deprived | - Lower consultations - Less cancer-related therapy - Less likely to see a radiation/medical oncologist after dx | - Material deprivation (Ontario Marginalisation Index Database Quintile 1-5) | Receipt of cancer care:   - Oncology consultation within 12 months of dx (Yes/No) - Receipt of cancer-related therapy (1 or more days of chemotherapy or radiotherapy within 12 months (Yes/No) | - Increased access to family physicians - Improved communication between family physicians and oncologists - Automatic referral systems - Provision of true patient-centered care - Screening patients for social determinants of health at presentation |
| Shah et al | The impact of socioeconomic status on stage at presentation, receipt of diagnostic imaging, receipt of treatment and overall survival in colorectal cancer patients | 2021 | Ontario (Canada) | Population-based registry | Cohort | 52,966 | Colorectum | Not provided | Lower SES | - Higher stage at presentation - Decreased receipt imaging (abdominal MRI) - Decreased receipt of surgery (liver and colon resection) | - Income quintile (1 lowest-5 highest) | - Stage at presentation (1-4) - Receipt of imaging (Yes/No) - Receipt of treatment (Surgery, Chemotherapy; Yes/No) | No |
| Noel et al | Association of Household Income at Diagnosis with Financial Toxicity,  Health Utility, and Survival in Patients With Head and Neck Cancer | 2022 | Canada | Teaching hospital | Cohort | 555 | Head & Neck | Not provided | Lowest income quartile | - Worse disease-free survival - Worse financial toxicity - Worse health status | - Annual household income at time of diagnosis (Income quartile) | - Disease-free survival (Months) - Subjective financial toxicity (FIT tool) - Health Utility (Health Utilities Index Mark 3) | No |
| Doumouras et al | Neighbourhood Variation in the Utilization of Laparoscopy for the Treatment of Colon Cancer | 2016 | Canada | Population-based multi-hospital database | Cohort | 9,969 | Colon | Not provided | - Rural neighbourhoods - Regions with less MIS training centres | - Less likely to receive laparoscopic colectomy (Rural neighbourhoods) - Less utilisation of MIS (Regions with less MIS training centres) | - Neighbourhood (Cold, Neutral, Hot clusters) | - Neighbourhood rates of laparoscopy | No |
| Fond et al | End of life breast cancer care in women with severe mental illnesses | 2021 | France | Population-based database | Cohort | 38,612 | Breast | Not provided | Women with severe psychiatric disorders (SPDs) | - More frequently hospitalised during their last month of life - Women with died earlier than those with recurrent major depression and without SPDs | - Women with severe psychiatric disorders (Diagnosed with at least one disorder: bipolar disorder, recurrent major depression, schizophrenia). | Palliative care indicators:   - Access to palliative care in the past 31 days (Y/N) - Duration from first palliative care and death (days)   High-intensity palliative care indicators: (Y/N)   - Intra-hospital chemotherapy in last 14 days of life, mechanical ventilation, blood transfusion, surgery, imaging, or endoscopy, at least one emergency department visit or ICU admission or one admission into an acute care unit in final 31 days of life | No |
| Scanlon et al | Exploring equity in cancer treatment,  survivorship, and service utilisation  for culturally and linguistically diverse migrant  populations living in Queensland, Australia:  a retrospective cohort study | 2023 | Australia | Teaching hospital | Cohort | 523 | All | “Health inequities are systematic, unfair, and avoidable differences in the opportunity’s groups have to attain optimal health and health  outcomes” | Culturally and Linguistically (CALD) migrants | - Delayed time from dx to commencement of first tx (radiation, surgery) - More likely to decline a recommended chemotherapy - More disease progression - Higher unplanned admissions - Longer length of unplanned admissions - Higher failure to attend scheduled appointments | - CALD migrants (CALD vs Australian-born) | - Time from dx to commencement of first tx (months) - Declining recommended chemotherapy (Y/N) - Disease progression (Y/N) - Number of unplanned admissions (*n*) - Length of unplanned admissions (days) - Failure to attend appointments (*n*) | - Need for consistent and clear documentation of interpreter usage |
| Heiden et al | Access to Care Metrics in Stage I Lung  Cancer: Improved Access Is Associated  With Improved Survival | 2022 | United States | Veterans Affairs Cancer Registry | Cohort | 9749 | Lung | Not provided | - Black ethnicity - Higher area deprivation index score - Increased distance to hospital | - Less likely to meet all 4 quality measures (Black ethnicity, higher area deprivation index, increased distance to hospital) | - Ethnicity (Black, White, Other, Unknown) - Area deprivation index score (Q1-Q5) - Distance to treating facility (Q1Closest –Q5 Furthest) | Quality measures:   - Timely surgery (definitive surgery treatment within 12 weeks of radiographic diagnosis) - Receipt of PET imaging (undergoing imaging within 6 months before surgery) - Appropriate smoking management (those who smoked receiving smoking cessation pharmacotherapy (NRT, bupropion or varenicline) within 12 months prior to surgery - Receipt of PFTs (undergoing evaluation with spirometry within 6 months before surgery) | No |
| Norris et al | Socioeconomic Inequalities in Novel NSCLC  Treatments During the Era of Tumor Biomarker-  Guided Therapy: A Population-Based Cohort Study  in a Publicly Funded Health Care System | 2023 | England | Population-based registry | Cohort | 90,785 | Lung | Not provided | Higher deprivation | - Less likely to use any novel therapy | - Deprivation (IMD) | - Utilisation of novel anti-cancer therapy (molecular targeted therapy, biological therapy, or Immune checkpoint inhibitors; Y/N) | No |
| Shah et al | Rural vs urban inequalities in stage at diagnosis for lung cancer | 2021 | Saskatchewan (Canada) | Population-based registry | Cohort | 2972 | Lung | Not provided | Rural geographic residence | - Higher relative risk of advanced stage at dx | - Geographic residence (Urban vs Rural) | Stage at dx (1-4) | No |
| Ousseine et al | Health Literacy and Clinical Trial Participation in French Cancer Patients: A National Survey | 2022 | France | National Survey | Longitudinal Survey | 1954 | All | Not provided | Limited health literacy | - Fewer clinical trials invitations | - Functional health literacy (Single item literacy screener, Test of Functional Health Literacy in Adults) | - Clinical Trial Invitations (Self-reported) | - Patient education to improve clinical trial knowledge - Early distribution to the patient of a list of questions about the clinical trial in order to stimulate discussion with the physician when discussing possible participation - Simplifying information documents to make them easier to read and understand for low HL patients - Developing standardized patient decision aids to improve cancer trial recruitment - Creating patient guides to help patients with limited HL to better understand clinical trials |
| Berchuck et al | Association of Mental Health Treatment With Outcomes for US Veterans Diagnosed With Non–Small Cell Lung Cancer | 2020 | United States | Veterans Affairs Cancer Registry | Cohort | 55, 315 | Lung | Not provided | Those with mental health disorders who did not participate in mental health treatment program (MHTP) | - Higher likelihood late stage dx - Higher all-cause mortality - Higher lung cancer-specific mortality - Less likely to receive stage appropriate tx | - Mental health disorders (including schizophrenia, bipolar disorder, depressive disorder, posttraumatic stress disorder, and substance use disorder). | - Stage at dx (1-4) - Receipt of stage-appropriate tx (National Comprehensive Cancer Network Guidelines) - All-cause mortality - Cancer-specific mortality | No |
| Beckmann et al | Short-term outcomes after surgical resection for colorectal cancer in South Australia | 2016 | Australia | Population-based registry | Cohort | 3940 | Colorectum | Not provided | - Lower SES - Non-metropolitan areas | - Higher risk of postoperative complications (lower SES) - Higher risk of 30-day mortality (non-metropolitan patients) | - SES (Index of Socioeconomic Advantage and Disadvantage) - Place of residence (Accessibility/Remoteness Index) | - Postoperative complications (Y/N) - 30-day mortality | No |
| Uehara et al | Travel Time and Distance and Participation in Precision Oncology Trials at the National Cancer Center Hospital | 2023 | Japan | Teaching hospital | Cohort | 1127 | All | Not provided | Those with higher travel times | - Lower clinical trial participation | - Travel time (<120 minutes vs >120 minutes) | - Clinical trial participation | No |
| Sandoval et al | Magnitude and Temporal Variations of Socioeconomic Inequalities in the Quality of Life After Early Breast Cancer: Results From the Multicentric French CANTO Cohort | 2024 | France | Population-based registry | Cohort | 5915 | Breast | Not provided | Lower SES | - Inequalities in QoL increased over time | - SES (self-reported financial difficulties, income, and educational level) | - QoL (QLQ-C30 score at diagnosis and 2-year follow up) | No |
| Steele et al | The Impact of Age on Colorectal Cancer Incidence, Treatment, and Outcomes in an Equal-Access Health Care System | 2014 | United States | Population-based registry | Cohort | 7948 | Colorectum | Not provided | Young age at presentation (<50 years) | - More advanced stage - Higher recurrence | - Younger age at presentation (<50 years) | - Stage at presentation (1-4) - Overall recurrence (at 3 and 5 years) | No |
| Majano et al | Mental Health Morbidities and Time to Cancer Diagnosis Among Adults With Colon Cancer in England | 2022 | England | Population-based registry | Cohort | 3766 | Colorectum | Not provided | Those with mental health morbidity (MHM) recorded prior to cancer dx | - More red-flag symptoms in the 24 months prior to dx - Lower odds of fast-track referral - Those with red-flag symptoms and MHM experienced 2-fold longer median time to dx - Higher odds of emergency dx | - MHM recorded prior to dx (anxiety, depression, schizophrenia, bipolar disorder, alcohol addiction, anorexia, and bulimia) | - Fast-track specialist referral (2-week wait) - Time from colonoscopy to dx - Emergency dx (Route to dx) | No |
| Kim et al | Relationship between socioeconomic status and accessibility for endoscopic resection among gastric cancer patients: using National Health Insurance Cohort in Korea: poverty and endoscopic resection | 2017 | South Korea | Population-based database | Cohort | 1671 | Stomach | Not provided | Lowest income group | - Lower rate of endoscopic mucosal resection (EMR) treatment | - Individual-level income (National Health Insurance Premium) - Neighbourhood SES (Modified Townsend and Carstairs Index) | - Receipt of EMR tx (Y/N) | No |
| Levin et al | Ethnic disparity in cervical cancer stage at diagnosis: A retrospective study in an Israeli referral-center | 2022 | Israel | Teaching hospital | Cohort | 252 | Cervix | Not provided | Arab women in Israel | - Higher prevalence of advanced stage at dx | - Ethnicity (Arab vs Israeli women) | - Stage at dx (1-4) | No |
| van Leeuwen | Reoperation after breast-conserving surgery for cancer in Australia: statewide cohort study of linked hospital data | 2018 | Australia | Population-based electronic health records database | Cohort | 34, 458 | Breast | Not provided | - Non-metropolitan location - Operation at low-volume hospitals | - Higher rates of reoperation (non-metropolitan locations and low volume hospitals) | - Residential and hospital location (Metropolitan/Non-Metropolitan) - Surgical volume (<15, 15-49, 50+ annual average) | - Reoperation rates (*n*/%) | No |
| Rinder et al | Dynamic Projection of Medication Nonpersistence and Nonadherence Among Patients With Early Breast Cancer | 2024 | France | Population-based database | Cohort | 229, 695 | Breast | Not provided | - Older age (>70 years) - Low income | - Higher rates of non-persistence and nonadherence (older age, lower income) | - Age (years) - Income (Median tax income) | - Persistence (Taking medication for intended timeframe) - Adherence (MPR <80%) | - Integrating telemonitoring into oncology services |
| Adrien et al | Why are head and neck squamous cell carcinoma diagnosed so late? Influence of health care disparities and socio-economic factors | 2014 | France | Population-based multi-hospital database | Cross-sectional study | 690 | Head & Neck | Not provided | - Male sex - Difficulty accessing specialist services | - Associated with late stage dx (men, difficulty accessing specialist services) | - Sex (Male/Female) - Ease of accessing specialist (Ease vs difficult) | - Late stage dx (T3 or T4 tumour) | No |
| Mahar et al | The effect of a severe psychiatric illness on  colorectal cancer treatment and survival: A  population-based retrospective cohort study | 2020 | Canada | Population-based electronic health records database | Cohort | 24, 507 | Colorectum | Not provided | Individuals with severe psychiatric illness (SPI) | - Lower survival - Less likely to receive guideline recommended treatment - Less likely to receive potentially curative surgical resection - Less likely to receive adjuvant radiation or chemotherapy | SPI history (No mental illness, outpatient SPI history, inpatient SPI history) | - Cancer-specific survival - Non-receipt of surgical resection - Non-receipt of adjuvant chemotherapy - Non-receipt of adjuvant radiation | No |
| Malhotra et al | Financial difficulties are associated with greater total pain and suffering among patients with advanced cancer: results from the COMPASS study | 2020 | Singapore | Multiple teaching hospitals | Cohort | 600 | Stage-4 Solid Malignancy | “The Universal Health Coverage goals call for access to affordable palliative care to reduce inequities in “total pain” and suffering”. | Patients with higher financial difficulties | - Worse physical, psychological, social, and spiritual outcomes - Lower perceived quality of health care coordination and responsiveness | Patient-reported financial difficulties (the extent to which their financial resources met their financial needs in relation to treatments, daily living expenses, and other obligations). Measured as: very well (= 1), fairly well (= 2), and poorly (= 3). | - Physical and functional wellbeing (FACT-G V4) - Pain severity and interference (Brief Pain Inventory) - Severity of other symptoms (FACIT-Pal) - Psychological outcomes (Hospital Anxiety and Depression scale) - Social wellbeing (FACT-G) - Spiritual outcomes (FACIT-Spiritual) - Perceived health care quality (Consumer Assessment of Healthcare Providers and Systems (adapted) | - Financial difficulties should directly be assessed in routine clinical settings among patients with advanced cancer rather than relying on routinely collected socio-economic indicators as proxy measures |
| Eaglehouse et al | Racial Differences in Time to Breast Cancer Surgery  and Overall Survival in the US Military Health System | 2019 | United States | Population-based registry | Cohort | 4887 | Breast | Not provided | Non-Hispanic Black Women | - Significantly greater estimated time to surgery - Higher adjusted risk for death during breast conserving surgery | Ethnicity (Non-Hispanic Black women vs Non-Hispanic White Women) | - Time to surgery (difference in days between the diagnosis and first surgery dates) - Overall survival (%) | No |
| Yamoah et al | Racial and Ethnic Disparities in Prostate Cancer Outcomes in the Veterans Affairs  Health Care System | 2022 | United States | Population-based registry | Cohort | 7,889, 984 | Prostate | Not provided | African American ethnicity | - Younger age at dx - Higher PSA levels at dx - Nearly 2 fold greater incidence of localised and de novo metastatic Pca at dx - Higher residual metastatic burden | Ethnicity (African American vs White race) | - Time to distant metastasis following Pca dx (days) - Age (years) - Time to treatment (days) - PSA levels (ng/mL) - Residual Metastatic Burden (Rate of metastasis of those receiving definitive Tx) | No |
| Zhang et al | Socioeconomic inequality in health care use among cancer patients in China: Evidence from the China health and retirement longitudinal study | 2022 | China | Longitudinal Survey | Cohort | 392 | All | Not provided | Low SES | - Less likely to use health services | Socio-economic status (annual per capita household expenditure) | - Outpatient service use (%) - Inpatient service use (%) | No |
| Cerni et al | Does Geography Play a Role in the Receipt of End-of-Life Care for Advanced Cancer Patients? Evidence from an Australian Local Health District Population-Based Study | 2022 | Australia | Population-based database | Cohort | 3546 | All (Advanced) | Not provided | Rural and regional patients  Longer travel times | - Higher ED visits (rural) - Higher ICU admissions (rural) - Lower rates of outpatient chemotherapy and radiotherapy (rural and regional) - Lower inpatient specialist palliative care (Longer travel times) | Geographic remoteness (Modified Monash Index and Travel time (Minutes) | - ED visits in last year of life (*n*) - ICU admissions in last year of life (*n*) - Outpatient chemotherapy in last year of life (*n*) - Outpatient radiotherapy in last year of life (*n*) | No |
| Lin et al | Geographical disparities in the prognosis of patients with nasopharyngeal carcinoma treated with intensity-modulated radiation therapy: a large institution-based cohort study from an endemic area | 2020 | China | Teaching hospital | Cohort | 1002 | Nasopharynx | Not provided | Rural patients | - Worse overall survival - Worse DFS - Worse locoregional relapse-free survival - Worse distant metastasis-free survival | Rurality (Rural vs Urban) | - Overall survival - DFS - Relapse-Free survival - Distant metastasis-free survival | No |
| Bergqvis et al | Equal receipt of specialized palliative care in breast and prostate cancer: a register study | 2022 | Sweden | Population-based registry | Cohort | 2516 | Breast, prostate | Not provided | - Older patients - Lower SES - Those with higher comorbidities | - Lower receipt of palliative care 3 months before death (Older patients, low SES, higher comorbidities) - Higher use of ED in 3 months before death (Older patients, low SES, higher comorbidities) - More likely to die in hospital (lower SES) | - Age (Years) - SES (Mosaic Group 1-3) - Comorbidities (CCI) | - Receipt of Specialist Palliative Care - ED visits (*n*) - Hospital as place of death (*n*) | No |
| Servadio et al | Investigating socioeconomic disparities in lung cancer diagnosis, treatment and mortality: an Italian cohort study | 2024 | Italy | Population-based database | Cohort | 13,251 | Lung | Not provided | Low socioeconomic position | - Higher risk of delayed diagnosis - Higher risk of mortality - Less access to advanced treatments for metastatic patients | Socioeconomic position (Level of education) | - Delayed diagnosis (if their first hospitalisation for lung cancer was preceded by an emergency room admission) - Mortality within the two years following diagnosis - Access to advanced drug therapies for lung cancer within the two years following diagnosis | No |
| Dickens et al | Stage at breast cancer diagnosis and distance from diagnostic hospital in a periurban setting: A South African public hospital case series of over 1,000 women | 2014 | South Africa | Teaching hospital | Case series | 1,071 | Breast | Not provided | >20 km from diagnostic hospital | - More late stage at dx | Distance from diagnostic hospital | - Stage at dx (Stage 1-4) | No |
| Souza et al | Factors associated with time to initiate lung cancer treatment in Minas Gerais, Brazil | 2022 | Brazil | Population-based database | Cohort | 4, 789 | Lung | Not provided | - Women - Older age - Living in regions with no oncology services | Less likely to initiate tx within 60 days of dx | - Sex (Male/Female) - Age (Years) - Macro-region of residence | Initiated tx within 60 days of dx (Yes/No) | No |
| Boyle et al | Disparities in time to breast cancer surgery in New Zealand by level of neighborhood deprivation: a population‑based study | 2025 | New Zealand | Population-based registry | Cohort | 20, 322 | Breast | Not provided | - Higher deprivation | - Higher delays to surgery | Deprivation (NZ Deprivation Index) | - Time from dx to surgery (days) | - Use of Clinical Priority Assessment Criteria for surgical waitlists - Increased Cancer Nurse Specialists |
| Choi et al | Disparities in the Diagnosis and Treatment of Breast Cancer Among People With Disabilities | 2025 | South Korea | Population-based database | Cohort | 150, 412 | Breast | “… barriers to timely and appropriate cancer diagnosis and care, resulting in poorer prognoses and higher mortality…include physical accessibility issues, biases among health care practitioners… and socioeconomic disadvantage… which collectively hinder equitable access to timely screening, diagnosis, and treatment”. | People with disabilities | - Higher stage at dx - Higher unknown stage at dx - Less likely to receive Tx (surgery, CTX or XRT) - Higher all-cause mortality - Lower 5-year survival | Disability status (physical, brain, sensory or additional impairments) | - Stage at dx (localised, locoregional, distant, unknown) - Receipt of standard treatments: (surgery Y/N, chemotherapy Y/N, Radiotherapy Y/N) - All-cause mortality (%) - 5-year survival | No |
| Pickwell-Smith et al | Are there inequalities in ovarian cancer diagnosis and treatment in England? A population-based study | 2025 | England | Population-based registry | Cohort | 9572 | Ovarian | Not provided | Higher deprivation | - Lower receipt of tx (Surgery and CTX) - Longer time to tx commencement | Deprivation (Index of Multiple Deprivation) | - Receipt of tx: (Surgery Y/N, CTX Y/N) - Secondary care diagnostic interval (days) - Treatment interval (days) - Whole interval (days) | - Referring patients for pre-rehabilitation - Personalising communication - Ensuring awareness of available financial and transportation support |
| Bondzi-Simpson et al | Association of neighborhood socioeconomic status and ethnic diversity with receipt of adjuvant chemotherapy in stage III colon cancer: A populationbased cohort study | 2025 | Canada | Population-based registry | Cohort | 14, 511 | Colon | Social determinants of health are formed by social structures and economic systems that can result in unequal access to power and resources that influence health and health outcomes | - Lower SES - Neighbourhood Ethnic diversity | - Lower odds of adjuvant CTX | - SES (Material Deprivation) - Neighbourhood Ethnic diversity (proportion of recent immigrants, non-white and non-indigenous populations) | - Receipt of CTX (within 3 months of surgery) | - Providing culturally-competent health information - Providers screen for social determinants of health and poverty using validated tools |

Diagnosis: Dx. Treatment: Tx. Socioeconomic Status: SES. Minimally invasive surgery: MIS. Medical Possession Ratio: MPR. Prostate Specific Antigen: PSA. Prostate Cancer: Pca. Emergency Department: ED. Disease-Free Survival: DFS. Chemotherapy: CTX. Radiation therapy: XRT.

* Only outcomes where inequities were demonstrated (P<0.05) were included  
** Only outcomes associated with clinical services were included
