# Supplementary material for: How equity in cancer services has been defined and measured, and why it matters: a scoping review of Universal Health Coverage systems
Source: Glob Health Action. 2026 Sep 16;19(1):2733814. doi: 10.1080/16549716.2026.2733814 (PMC13587566; doi:10.1080/16549716.2026.2733814)
Supplement: Supplementary file 3 [file ZGHA_A_2733814_SM3648.docx]

**Supplementary File 1: Database Search Strategy**

**Title:** *How equity in cancer care has been defined and measured and why it matters: A systematic scoping review of Universal Health Coverage Systems*

Search strategy includes key concepts and limits:

(equity) AND (cancer) AND (methodological limits) AND (last 10 years) AND (limited to Humans)

**Database searches**

Initial date searched – 1 November 2024

Search repeated – 17 November 2025

**PubMed** 1446 results

Includes MeSH

("Health Equity"[MeSH] OR "Healthcare Disparities"[MeSH] OR “equity”[tiab] OR “equities”[tiab] OR “equitable”[tiab] OR “inequity”[tiab] OR “inequities”[tiab] OR “inequitable”[tiab] OR “inequality”[tiab] OR “inequalities”[tiab] OR “equality”[tiab] OR “equalities”[tiab] OR “disparity”[tiab] OR “disparities”[tiab] OR “underserved”[tiab]) AND ("Neoplasms"[MeSH] OR "Medical Oncology"[MeSH] OR "Oncology Nursing"[Mesh] OR "Surgical Oncology"[Mesh] OR "Oncology Service, Hospital"[Mesh] OR "Cancer Care Facilities"[Mesh] OR “cancer”[tiab] OR “cancers”[tiab] OR “oncology”[tiab] OR “oncological”[tiab] OR “tumors”[tiab] OR “tumours”[tiab] OR “tumor”[tiab] OR “tumors”[tiab] OR “neoplasia”[tiab] OR “neoplasias”[tiab] OR “neoplasm”[tiab] OR “neoplasms”[tiab] OR “malignant”[tiab] OR “malignancy”[tiab] OR “malignancies”[tiab]) AND (“measure”[tiab] OR “measures”[tiab] OR “measured”[tiab] OR “measuring”[tiab] OR “indicator”[tiab] OR “indicators”[tiab]) AND ("Clinical Studies as Topic"[Mesh] OR "Clinical Trials as Topic"[Mesh] OR "Clinical Study" [Publication Type] OR "Cohort Studies"[Mesh] OR "Controlled Before-After Studies"[Mesh] OR "Historically Controlled Study"[Mesh] OR "randomized"[tiab] OR "randomised"[tiab] OR "randomly"[tiab] OR "trial"[tiab] OR “cohort”[tiab]) AND 2014:2025[dp] NOT (animals [mh] NOT humans [mh])

**CINAHL Complete (EBSCOhost)** 504 results

Includes CINAHL Subject Headings

(MH "Healthcare Disparities" OR TI(“equity” OR “equities” OR “equitable” OR “inequity” OR “inequities” OR “inequitable” OR “inequality” OR “inequalities” OR “equality” OR “equalities” OR “disparity” OR “disparities” OR “underserved”) OR AB(“equity” OR “equities” OR “equitable” OR “inequity” OR “inequities” OR “inequitable” OR “inequality” OR “inequalities” OR “equality” OR “equalities” OR “disparity” OR “disparities” OR “underserved”)) AND (MH "Neoplasms+" OR MH "Oncology+" OR MH "Oncologic Care+" OR MH "Oncology Nursing+" OR MH "Oncology Surgery+" OR MH "Cancer Care Facilities" OR MH "Oncology Care Units" OR MH "Cancer Patients" OR TI(“cancer” OR “cancers” OR “oncology” OR “oncological” OR “tumors” OR “tumours” OR “tumor” OR “tumors” OR “neoplasia” OR “neoplasias” OR “neoplasm” OR “neoplasms” OR “malignant” OR “malignancy” OR “malignancies”) OR AB(“cancer” OR “cancers” OR “oncology” OR “oncological” OR “tumors” OR “tumours” OR “tumor” OR “tumors” OR “neoplasia” OR “neoplasias” OR “neoplasm” OR “neoplasms” OR “malignant” OR “malignancy” OR “malignancies”)) AND (TI(“measure” OR “measures” OR “measured” OR “measuring” OR “indicator” OR “indicators”) OR AB(“measure” OR “measures” OR “measured” OR “measuring” OR “indicator” OR “indicators”)) AND (MH "Clinical Trials+" OR MH "Prospective Studies+" OR MH "Controlled Before-After Studies" OR MH "Historically Controlled Study" OR MH "Nonrandomized Trials" OR TI("randomized" OR "randomised" OR "randomly" OR "trial" OR “cohort”) OR AB("randomized" OR "randomised" OR "randomly" OR "trial" OR “cohort”)) AND PY 2014-2025 NOT ((MH "Animals+" OR MH "Animal Studies" OR TI animal model*) NOT MH "Human")

*Link to saved Cochrane search -* [*https://www.cochranelibrary.com/advanced-search/search-manager?search=7594578*](https://www.cochranelibrary.com/advanced-search/search-manager?search=7594578)

**Cochrane Library (Wiley) – CENTRAL** 473 Trials

Includes MeSH

ID	Search	Hits

#1	MeSH descriptor: [Health Equity] explode all trees	33

#2	MeSH descriptor: [Healthcare Disparities] explode all trees	346

#3	(“equity” OR “equities” OR “equitable” OR “inequity” OR “inequities” OR “inequitable” OR “inequality” OR “inequalities” OR “equality” OR “equalities” OR “disparity” OR “disparities” OR “underserved”):ti,ab,kw	11070

#4	#1 OR #2 OR #3	11070

#5	MeSH descriptor: [Neoplasms] explode all trees	127535

#6	MeSH descriptor: [Medical Oncology] explode all trees	628

#7	MeSH descriptor: [Oncology Nursing] explode all trees	227

#8	MeSH descriptor: [Surgical Oncology] explode all trees	10

#9	MeSH descriptor: [Oncology Service, Hospital] explode all trees	27

#10	MeSH descriptor: [Cancer Care Facilities] explode all trees	125

#11	(“cancer” OR “cancers” OR “oncology” OR “oncological” OR “tumors” OR “tumours” OR “tumor” OR “tumors” OR “neoplasia” OR “neoplasias” OR “neoplasm” OR “neoplasms” OR “malignant” OR “malignancy” OR “malignancies”):ti,ab,kw	283782

#12	#5 OR #6 OR #7 OR #8 OR #9 OR #10 OR #11	296695

#13	(“measure” OR “measures” OR “measured” OR “measuring” OR “indicator” OR “indicators”):ti,ab,kw	502336

#14	#4 AND #12 AND #13 with Publication Year from 2014 to present, in Trials	473
